# The relationship between internet use preference and loneliness among college students during COVID-19: The chain mediating effect of online social support and self-esteem

**DOI:** 10.3389/fpsyg.2022.1058944

**Published:** 2022-12-22

**Authors:** Qing Luo, Lu Huang, Na Wu

**Affiliations:** ^1^Department of Psychology, School of Public Policy and Administration, Nanchang University, Nanchang, China; ^2^School of Marxism, Wuhan Business University, Wuhan, China; ^3^Department of Psychology, College of Education and Science, Hubei Normal University, Huangshi, China

**Keywords:** internet use preference, loneliness, online social support, self-esteem, chain mediating effect

## Abstract

The outbreak of COVID-19, especially the demands of social interaction and spatial distancing behavior, has led to a surge in Internet use, which has also led to an increase in loneliness. Therefore, we investigated the role of online social support and self-esteem in the relationship between Internet use preference and loneliness. In this study, 1053 college students were surveyed with a questionnaire based on the framework of Ecological System Theory, and a chain mediation model was established to clarify the mechanism between Internet use preference and loneliness. The results show that Internet use preference not only positively predicts loneliness, but also indirectly influences loneliness through the mediators of online social support and self-esteem, thereby impacting loneliness through the “online social support → self-esteem” chain. The results also indicate the need to pay attention to college students’ mental health status during COVID-19. The advent of COVID-19 has impacted people’s lifestyles and has changed the impact of the Internet on individual mental health. This study provides a new way to further understand college students’ Internet use preferences, online social support, self-esteem, and loneliness status during COVID-19. It provides targeted interventions for college students’ loneliness during COVID-19.

## 1. Introduction

COVID-19 has had a huge impact on people’s lives, especially the mental health of individuals (Zandifar and Badrfam, 2020). New demands have been placed on people’s social interactions and spatial distance behaviors during COVID-19, leading to a surge in Internet use (King et al., 2020; Mestre-Bach et al., 2020). Adolescents have reported higher Internet use amid the “new normal” pandemic era as compared to before (Singh et al., 2021). Classroom teaching moved from offline to online; face-to-face meetings became online meetings; office locations became telecommuting from home; and even connecting with friends, obtaining information, and so on is done online (Carroll and Conboy, 2020; Dwivedi et al., 2020; Guessoum et al., 2020; Fernandes et al., 2021). Accordingly, web developers have launched a variety of web platforms to meet user requirements. Users can send and receive emails, software applications (Greenstein, 2020), conduct business and service transactions, access information and knowledge (Salemink et al., 2017; Goncalves et al., 2018), and participated in interpersonal communication and interaction (Greenstein, 2020) *via* the Internet.

During the first half of 2022, all types of personal Internet applications continued to grow in China, with the number one user group pertaining to basic applications (e.g., instant messaging, search engines, online entertainment; China Internet network information center, 2022). What could be done “in person” before can now only be done through the Internet, which has become a necessity in people’s lives. Ecological System Theory suggests that individuals develop by interacting in ecosystems (Bronfenbrenner, 1979) and that it is important to examine the significance of individual characteristics in the context (Sira and Ballard, 2009). Mental health problems brought about by COVID-19 (Holmes et al., 2020; Kola, 2020; Torales et al., 2020) may be related to the Internet use (Sun et al., 2020). Activities such as learning, working, and entertaining *via* the Internet are not the same as spending time and interacting with classmates, family, and colleagues offline, and long-term Internet use may lead to a rise in loneliness and have impact individuals’ mental health (Marsden et al., 2020; Taquet et al., 2021). Some individuals affected by COVID-19 have experienced social disconnection, accompanied by a perceived lack of social support and loneliness (Smith and Lim, 2020; Pai and Vella, 2021).

Loneliness is a negative social and emotional experience of feeling unacceptable or unpleasant about the lack of human relationships (Cunningham et al., 2021; Sipowicz et al., 2021). Loneliness is a common consequence of social restrictions associated with COVID-19 (Cunningham et al., 2021; Wallinheimo and Evans, 2022), and is generally used as an indicator of mental health when exploring the relationship between individual mental health and Internet use (Kraut et al., 1998; Deng and Wang, 2005). Loneliness is experienced throughout the lifespan but peaks in early adulthood (Shovestul et al., 2020). It is typical and common among college students, with important impacts on their mental health (Zhang et al., 2012). Loneliness affects an individual’s physical and mental health (Groarke et al., 2020; Jeste et al., 2020). Therefore, many researchers have called for a study of loneliness during the COVID-19 pandemic (Holmes et al., 2020; Smith and Lim, 2020).

As described by the Uses and Gratifications Theory, Internet use is goal-oriented and it can meet individual needs (Katz and Blumler, 1974; Falgoust et al., 2022). However, the academic literature has yielded inconsistent findings concerning Internet use and loneliness. While some studies have found that Internet use increases loneliness (Casale and Fioravanti, 2011; Romer et al., 2013; Jia et al., 2018), others have shown the exact opposite (Amichai-Hamburger and Hayat, 2011; Shen et al., 2013). The displacement hypothesis of Internet use suggests that individuals spend a lot of time on the Internet and less time on offline activities, leading to increased levels of loneliness (Kraut et al., 1998; Nie, 2001). The inconsistency of the results suggests that the relationship between Internet use and loneliness needs to be studied in depth to further understand the mechanisms between the two variables. Some studies suggest that these discrepancies may be influenced by the content of Internet activities (Selfhout et al., 2009; Zhou and Wang, 2015).

Researchers have classified Internet use services or content differently (Swickert et al., 2002; Kaynar and Amichai-Hamburger, 2008). Lei and Liu (2005) classified Internet usage preferences into four categories—information, transaction, entertainment and social—based on the findings of China Internet network information center (2007). Users use different Internet services and features for different motivations and needs, and this individual behavior of frequent and repeated use of certain Internet services and features is known as Internet usage preference (Li et al., 2012). The preference for online social interaction is positively associated with loneliness (Leung, 2011; Ye and Lin, 2015), and the preference for online social interaction could positively predict loneliness (Chen, 2019). Cell phone use preferences positively predict loneliness (Huang, 2018), SoLoMo (a social tool) use preferences are significantly positively associated with loneliness (Shen, 2013), and Internet social preferences can positively predict loneliness (He, 2018). Morgan et al. (2003) also found that interaction-oriented Internet use increased mental health, while non-interaction-oriented Internet use decreased mental health, and Internet social preferences were strongly associated with loneliness (Caplan, 2003). However, some researchers found that both interaction-oriented and non-communication-oriented Internet use reduces individuals’ social participation in real life, which leads to increased loneliness (Kraut et al., 1998).

Social support, as an external influence, is one of the best predictors of loneliness and plays an important role in human psychological well-being (Kong and You, 2013). In investigating the relationship between Internet use and loneliness, online social support, and social support can be used as indicators of social integration at the individual level (Weiser, 2001). Social support is a negative predictor of loneliness (Gökhan, 2010; Kong and You, 2013). The lack of social support is associated with higher loneliness, while adequate social support is associated with lower loneliness (Ellwardt et al., 2013). Social provisions theory assumes that individuals seek specific social support functions *via* different relationships with others and that different types of relationships provide different social support functions and meet different social needs (Weiss, 1974). Individuals can obtain social support through different types of online services (Lee and Cho, 2019; Shi et al., 2019). Individuals are likely to find peers with common interests and views through the Internet, thus gaining more social support and reducing loneliness (Amichai-Hamburger and Hayat, 2011; Tian et al., 2021). College students who have high levels of online social support are likely to spend more time on the Internet (Brailovskaia et al., 2019; Fang et al., 2020). Shaw and Gant (2002) found that Internet use led to a significant decrease in loneliness when combined with a significant increase in perceived social support. The study found that online social support from Facebook wan effective in reducing loneliness (Gilmour et al., 2019).

Self-esteem is an important factor to consider when exploring the situation of individuals in the context of the COVID-19 pandemic (Tsai, 2022). Self-esteem reflects the difference between an individual’s perceived real and ideal or desired state of self (Rosenberg, 2015). Self-esteem is an important factor in societal adaptation; it mediates the relationship between individuals and their environment and plays an important role in human communication (Greenberg et al., 1992). The Internet can meet self-esteem needs during adolescence (Liang, 2008), different types of Internet use are associated with self-esteem (Mitchell, 1999), and online social media could even help predict self-esteem (Ni and Shao, 2019). Similarly, Valkenburg et al. (2006) confirmed that adolescents could increase their self-esteem through good social feedback *via* social networks and that individuals with low self-esteem could obtain more social resources from the Internet. Moreover, self-esteem is an important predictor of loneliness (Jackson et al., 2000), with multiple studies showing evidence of a significant negative relationship (Skues et al., 2012; Kong and You, 2013). Self-esteem and loneliness are mutually reinforcing over time (Perlman and Peplau, 1981), including a long-term recurrent effect (Vanhalst et al., 2013). Low self-esteem and high loneliness are associated with social media addiction, which implies frequent use of Internet social features; that is, a preference for Internet social features (Andreassen et al., 2012). Self-esteem played a mediating role between social networking sites use and loneliness (Ye, 2019; Lin et al., 2022). Given the multiple characteristics of self-esteem (e.g., driven, social, and regulatory), it works as a mediator between various psychological variables (Zhang et al., 2004).

Finally, Social support is an important part of maintaining individuals’ self-esteem (Zhang et al., 2022). Social support and self-esteem have important protective effects on adolescent mental health (Yang and Ye, 2014; Orth et al., 2016), and these factors jointly influence the mental health of college students (Wang, 2018). The main-effect model of social support suggests that social support not only directly affects loneliness but also imposes indirect effects through individual psychological states and other relevant factors (Uchino et al., 1996). The use of social features of the Internet is sometimes stressful and may threaten individuals’ perceptions of social support (Trepte et al., 2015), which can lead to lower levels of self-esteem (Haferkamp and Krämer, 2011). The failure to receive expected social support may result in lower self-esteem (Li et al., 2018). Perceived social support and self-esteem sequentially mediate the relationship between active social networking sites use and loneliness (Lin et al., 2022). One study showed that information support, peer support, and instrumental support in online social support were direct negative predictors of self-esteem and could increase loneliness (Liang, 2008).

**Figure 1:**
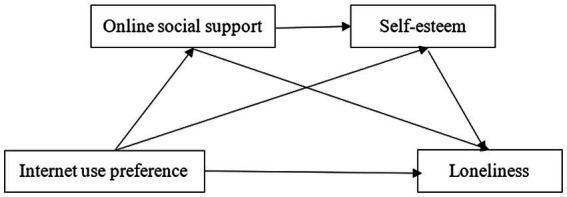
Model of the chain mediating roles of online social support and self-esteem.

In summary, based on previous studies and Ecological System Theory (Bronfenbrenner, 1979), this study explored the underlying mechanisms in the relationship between Internet use preference and loneliness. We constructed a research model with Internet use preference as the independent variable, loneliness as the dependent variable, and online social support and self-esteem as mediating variables (Figure 1). We proposed the following four hypotheses:

*Hypothesis 1*: Internet use preference can positively predict loneliness.

*Hypothesis 2*: Online social support mediates the relationship between Internet use preference and loneliness.

*Hypothesis 3*: Self-esteem is a mediating variable in the relationship between Internet use preference and loneliness.

*Hypothesis 4*: Online social support and self-esteem have a chain mediating effect in the relationship between Internet use preference and loneliness.

## 2. Materials and methods

### 2.1. Participants

This study conducted a survey among a convenience sample of 1,200 undergraduate students from four universities in central China. A total of 147 participants were excluded from the questionnaire because of short response times (less than 30 s) or contradictory answers (e.g., for the question, “I tend to feel that I am a failure,” the answer was “totally agree;” and to the question, “I am positive about myself,” the answer was “totally agree”). Finally, 1,053 valid questionnaires were obtained (return rate of 87.75%). Of these respondents, 685 (65.1%) were women and 368 (34.90%) were men, including 229 (21.70%) freshmen, 380 (36.1%) sophomores, 228 (21.7%) juniors, and 216 (20.5%) seniors. The average age was 19.46 ± 1.53 years.

### 2.2. Measures

#### 2.2.1. Adolescent internet service usage preference questionnaire

This study measured Internet service use preference *via* the Adolescent Internet Service Usage Preference Questionnaire (Lei and Liu, 2005), which consists of 17 items across four dimensions, including information, transaction, entertainment, and social. All items are answered on a five-point Likert scale ranging from 1 (*dislike very much*) to 5 (*like very much*). The sum of the scores of each item is the total score, and the higher the total score, the stronger the individual’s preference for Internet use. In this study, the scale received a Cronbach’s alpha coefficient of 0.83, AVE = 0.61, CR = 0.83. The fit indices of the scale are as follows: *χ^2^/df =* 3.74, *RESEA =* 0.04, *TLI =* 0.95, *CFI =* 0.96, indicating that the scale fits well.

#### 2.2.2. UCLA loneliness scale

This study measured loneliness *via* the third edition of the UCLA Loneliness Scale developed by Wang et al. (1999) which consists of 20 items, including 11 “loneliness” positive-order items and nine “non-loneliness” negative-order items. Answers are given on a four-point Likert scale. The sum of the scores of each item is the total score, such that higher total scores indicate higher levels of loneliness. In this study, the scale received a Cronbach’s alpha coefficient of 0.82, AVE = 0.63, CR = 0.83. The fitted metrics of the one-factor structural model are as follows: *χ*^2^*/df =* 3.39, *RESEA =* 0.06, *TLI =* 0.96, *CFI =* 0.93, indicating that the scale fits well.

#### 2.2.3. Adolescent online social support questionnaire

This study measured online social support using the adolescent online social support questionnaire developed by Liang (2008), which consists of 23 items across four dimensions, including information support, peer support, emotional support, and instrumental support. Answers are given on a five-point Likert scale ranging from 1 (*not at all*) to 5 (*completely*). The sum of the scores of each item is the total score, with higher scores indicating more online social support. In this study, the scale received a Cronbach’s alpha coefficient of 0.88, AVE = 0.51, CR = 0.90. The fit indices of the scale are as follows: *χ^2^/df =* 1.74, *RESEA =* 0.06, *TLI =* 0.90, *CFI =* 0.98, indicating that the scale fits well.

#### 2.2.4. Self-esteem scale

This study measured self-esteem *via* the self-esteem scale developed by Rosenberg (1965), which contains 10 items, including five that are positively scored and five that are negatively scored. These items are rated on a four-point Likert scale ranging from “very unconforming” to “very conforming.” The total score is obtained by adding the scores of each item after the reverse scoring, such that higher the total mean scores indicate higher levels of self-esteem. In this study, the scale received a Cronbach’s alpha coefficient of 0.81, AVE = 0. 55, CR = 0.85. The fitted metrics of the one-factor structural model are as follows: *χ*^2^*/df =* 4.56, *RESEA =* 0.06, *TLI =* 0.92, *CFI =* 0.96, indicating that the scale fits well.

### 2.3. Procedure and data processing

Prior to distribution, three psychology and cyberpsychology experts were invited to evaluate the questionnaires, thus ensuring that the contents would not affect participants. Participants were then invited *via* WeChat and QQ and completed the questionnaire *via* Wenjuanxing’s platform. In all cases, we first assured participants of privacy and confidentiality, obtained their informed consent, clearly articulated the instructions, and explained that there were no right or wrong answers.

Data were collected between March and July 2022. The collected data were subjected to a stepwise analysis with the aid of statistical software, including IBM SPSS 25.0 and PROCESS. First, we used Harman’s one-way test for common method bias for the original data; second, descriptive statistics and correlation analysis were performed on the data; then, to examine the chain mediating effect, we used Model 6 in the SPSS macro developed by Hayes (2017) to test the theoretical model, while controlling for sex (Fernandes et al., 2021). We estimated the 95% confidence intervals of the mediating effect with 5,000 resamples.

## 3. Results

### 3.1. Common method bias test

This study collected self-reported data, which may entail common method bias. To avoid this, corresponding controls were made concerning the procedures, including the use of anonymous responses, as suggested by Zhou and Long (2004). Then, the Harman one-way test was used to assess the raw data for common method bias. The results showed that 16 factors with eigenvalues greater than 1 were analyzed, with the first factor explaining 14.566% of the variance; this was less than the critical criterion of 40%, thus indicating no significant common method bias.

### 3.2. Descriptive and Pearson correlation results

Correlation analyses were conducted between Internet use preference, online social support, self-esteem, and loneliness. The results showed that there were significant positive correlations between Internet use preference and online social support, between Internet use preference and self-esteem, and between Internet use preference and loneliness; while there were significant negative correlations between loneliness and online social support, and between loneliness and self-esteem (Table 1).

**Table 1:** Correlations among variables, means, and standard deviations.

**Variables**	***M***	***SD***	**1**	**2**	**3**	**4**
1 Internet use preference	54.604	9.046	1			
2 Online social support	30.614	13.623	0.203^**^	1		
3 Self-esteem	29.162	4.183	0.159^**^	−0.070^*^	1	
4 Loneliness	53.125	6.352	0.067^*^	−0.113^*^	−0.116^**^	1

^*^p < 0.05, ^**^p < 0.01, ^***^p < 0.001.

### 3.3. Chain mediation model analysis

The regression analysis showed that Internet use preference significantly and positively predicted loneliness (*β* = 0.104, *p* < 0.01), online social support (*β* = 0.202, *p* < 0.001), and self-esteem (*β* = 0.179, *p* < 0.001), while online social support significantly and negatively predicted self-esteem (*β* = −0.098, *p* < 0.01). After adding the mediating variables, Internet use preference significantly and positively predicted loneliness (*β* = 0.067, *p* < 0.05), while online social support significantly and negatively predicted loneliness (*β* = −0.071, *p* < 0.05), and self-esteem significantly and negatively predicted loneliness (*β* = −0.142, *p* < 0.001; Table 2).

**Table 2:** Regression analysis on the relationships between variables.

**Regression equation**		**Overall fit index**			**Regression coefficient**	**Significance**
Result variables	Predictive variables	*R*	*R* ^2^	*F*	*β*	*t*
Loneliness	Internet use preference	0.094	0.009	4.666	0. 104	3.297^**^
Online social support	Internet use preference	0.265	0.071	39.790	0.202	6.785^***^
Self-esteem	Internet use preference	0.197	0.039	14.111	0.179	5.801^***^
	Online social support				−0.098	−3.109^**^
Loneliness	Internet use preference	0.177	0.031	8.436	0.067	2.184^*^
	Online social support				−0.071	−2.231^*^
	Self-esteem				−0.142	−4.579^***^

^*^p < 0.05, ^**^p < 0.01, ^***^p < 0.001.

**Figure 2:**
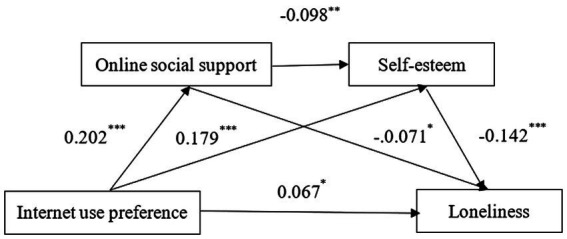
Chain-mediated pathway of online social support and self-esteem.

The mediation effect test showed that online social support and self-esteem mediated the effect of Internet use preference on loneliness, with a mediation effect value of −0.037, accounting for 55.06% of the total effect. The mediation effect consisted of indirect effects generated by three paths. Path 1 consisted of Internet use preference → online social support → loneliness, with a confidence interval that did not contain 0 ([−0.012, −0.003]), indicating that the indirect effect generated by this path was significant. Path 2 consisted of Internet use preference → online social support → self-esteem → loneliness, with a confidence interval that did not contain 0 ([0.001, 0.006]), indicating that the indirect effect generated by this path was significant. Path 3 consisted of Internet use preference → self-esteem → loneliness, with a confidence interval that did not contain 0 ([−0.044, −0.012]), indicating that the indirect effect generated by this path was significant (Table 3; Figure 2). Thus, the effect of Internet use preference on loneliness was achieved through the chain mediating effect of online social support and self-esteem as well as the separate mediating effects of each.

**Table 3:** Analysis of the mediating effects of online social support and self-esteem.

**Mediation path**	**Effect value**	**Boot CI lower**	**Boot CI upper**	**Effect ratio**
Internet use preference → online social support → loneliness	−0.014	−0.012	−0.003	21.39%
Internet use preference → online social support → self-esteem → loneliness	0.003	0.001	0.006	4.20%
Internet use preference → self-esteem → loneliness	−0.026	−0.044	−0.012	37.90%

## 4. Discussion

This study collected data from college students to explore the relationship between Internet use preference and loneliness during COVID-19, with a focus on clarifying the underlying mechanism. The results showed that Internet use preference not only directly affected loneliness but indirectly affected loneliness through the mediators of online social support and self-esteem. This mediating effect was realized through three pathways, including the (1) independent mediation of online social support, (2) independent mediation of self-esteem, and (3) chain mediating effect of online social support and self-esteem.

The finding that Internet use preference positively predicts loneliness in college students is consistent with existing findings (Chopik, 2016; Stockwell et al., 2021; Deutrom et al., 2022). This finding supports the displacement hypothesis of Internet use, wherein a substantial amount of the user’s time is allocated for such use, thus leaving less time for offline activities, which leads to increased feelings of isolation (Kraut et al., 1998; Nie, 2001). Particularly, during COVID-19, college students had to rely on the Internet for various activities owing to restrictions associated with COVID-19. In this context, this may lead to a situation in which the overly rich Internet features consume a lot of an individual’s time, thus reducing offline communication time with family and friends, narrowing the real social circle, and ultimately leading to an increase in users’ loneliness after being offline (Kraut et al., 1998; Liu, 2006). Contrastingly, during COVID-19, individuals can only passively perform various activities *via* the Internet, leading to increased levels of loneliness (Belfort and Miller, 2018).

The finding that online social support mediates the relationship between Internet use preference and loneliness in college students (i.e., Internet use preference can influence loneliness through online social support) supports the main-effect model of social support (Lakey and Cohen, 2000), which suggests a general gaining effect in which increased social support (regardless of current levels) improves individual mental health (e.g., loneliness; Barnum et al., 1998). Online social support is associated with lower negative outcomes (e.g., loneliness; Zhou and Cheng, 2022). This finding also provides further evidence that although online social support is a form that exists in cyberspace; it plays the same role as traditional social support (i.e., an increase in the level of online social support can also reduce the level of loneliness; Liu et al., 2022). In fact, online social support is considered a vital source of support for individuals during COVID-19. Various Internet functions (e.g., entertainment and communication) can bring more people together, thus providing opportunities for them to appreciate, support, and help each other (Tian et al., 2021). To some extent, this can reduce or alleviate loneliness and improve their mental health during COVID-19.

This study found that self-esteem mediates the relationship between Internet use preference and loneliness in college students (i.e., Internet use preference can influence loneliness through self-esteem). According to social identity theory (Tajfel and Turner, 1986), people automatically categorize things into in-groups and out-groups by means of positive distinctions that conform to the characteristics of the in-group given to the self, such that its behavior is consistent with the group. Internet use preference is in fact an in-group of individuals who see other individuals using the same function as an in-group when using different functions of the Internet, and then assign this characteristic to the self, which has the direct effect of raising the individual’s self-esteem level. Social cognitive theory also suggests that individuals with low self-esteem engage in certain behavioral and cognitive processes that hinder the development of social relationships and lead to increased loneliness (Perlman and Peplau, 1981; Brage et al., 1993). Therefore, loneliness decreases when the level of self-esteem increases.

Finally, this study found that Internet use preference influences loneliness in college students through the “online social support → self-esteem” chain of mediation. In the process of using various functions of the Internet, although college students can obtain online social support, it differs from the unpaid support they receive from their parents and friends in reality, and the social support provided by the Internet is not as useful as the support provided by friends in real life (Zhou and Cheng, 2022). Low self-esteem is often caused by the lack of unconditional support from parents and friends (Harter, 1993; Parker and Benson, 2004), and the social support obtained on the Internet is often conditional, especially during COVID-19; that is, people are more demanding in the online social support they need. The de-inhibited, physically absent nature of online social support may entail qualitative differences from the type of social support received in reality (Mickelson, 1997; Ding, 2003). Therefore, online social support that is not unconditionally available may lead to lower levels of self-esteem in individuals.

It could also be that social support on the Internet during COVID-19 was more negative, causing individuals to experience low self-esteem (Ho and Ito, 2019). In addition, according to the sociometric theory of self-esteem (Leary et al., 1995), self-esteem can reflect the emotional state of the degree that an individual is integrated into interpersonal relationships (i.e., self-esteem can reflect whether an individual has good interpersonal relationships, and thus good emotional experiences). Interpersonal relationships are largely influenced by the strength of online connections (Kraut et al., 1998). Online social support is weak, and virtual interpersonal relationships are difficult to maintain in real life. This makes it difficult for individuals to internalize the strengths gained from online social support, which leads to lower levels of self-esteem and consequently higher levels of loneliness. In sum, Internet use preference can influence loneliness through the “online social support → self-esteem” chain.

## 5. Conclusion

In this study, we explored the mechanisms of the role of the Internet on mental health in the COVID-19 context. The results revealed that Internet use preference not only directly affects loneliness but also has indirect effects through online social support, self-esteem, and online social support-self-esteem, respectively. Our study shows that the occurrence of COVID-19 alters the impact of the Internet on the mental health of college students. While connecting with other through the Internet was always an individual’s optional choice, it became more mandatory during the pandemic. These online social interactions may be detrimental to individuals’ psychological health when they replace face-to-face sociality.

The current findings have critical implications for policymakers. Schools and education departments need to actively focus on the impact of public health measures that restrict social interaction during COVID-19 on individual mental health (Cunningham et al., 2021). Schools and teachers should provide unconditional social support to students by focusing on feelings of loneliness owing to the new demands of social interaction and spatial distance. Education departments should be sympathetic to the dilemmas and psychological problems faced by college students in the general environment of COVID-19.

### 5.1. Limitations and future directions

This study had some limitations. First, this study only considered Internet use preferences and did not consider variables such as the duration of Internet use and specific preferences for each function, which may lead to a less comprehensive study of the impact of Internet use on mental health. Given the complexity of Internet platform functions and their complex effects on individual mental health, future studies should consider the integration and differentiation of Internet functions, such as studying the effects of various Internet functions on individuals from the perspectives of Internet developers and users, and examining the effects of active and passive Internet use on individuals. Second, all participants were from central region of China, and the survey schools were concentrated in central China; however, students came from all over the country. Thus, the results are representative; however, caution is needed in the generalization of the results. Future studies need to expand the sample group, not only by expanding the age of the participants to the younger and older age groups but also by expanding the survey area to other remote areas of China to improve the generalizability of the findings. Finally, this study only examined the mechanism of action between Internet use preference and loneliness through questionnaires and cross-sectional data; however, it is unclear whether the mechanism continues to act at different stages of an individual’s development. Therefore, future studies should use experimental methods and conduct longitudinal studies to analyze the association between Internet use preference and loneliness to support the causal relationship between variables with stronger arguments. In addition, the impact of Internet use on loneliness could be further explored to see whether the effect of Internet use on loneliness is consistent across different sexes and ages during the COVID-19 pandemic.

## Data availability statement

The raw data supporting the conclusions of this article will be made available by the authors, without undue reservation.

## Ethics statement

The studies involving human participants were reviewed and approved by Scientific Review Committee of the School of Public Policy and Administration, Nanchang University. The patients/participants provided their written informed consent to participate in this study.

## Author contributions

QL contributed to the experimental design, analyzed the data, and drafted the manuscript. LH helped to revise the manuscript. NW provided final approval of the manuscript. All authors contributed to the article and approved the submitted version.

## Funding

This study was supported by the Jiangxi Provincial Social Science Thirteenth Five-year Plan Planning Youth Project (grant no. 17JY32) and the Humanities and Social Sciences Research Planning Youth Project for Universities and Colleges in Jiangxi Province (grant no. XL18106).

## Conflict of interest

The authors declare that this research was conducted in the absence of any commercial or financial relationships that could be construed as a potential conflict of interest.

## Publisher’s note

All claims expressed in this article are solely those of the authors and do not necessarily represent those of their affiliated organizations, or those of the publisher, the editors and the reviewers. Any product that may be evaluated in this article, or claim that may be made by its manufacturer, is not guaranteed or endorsed by the publisher.

## References

[ref1] Amichai-Hamburger Y. Hayat Z. (2011). The impact of the internet on the social lives of users: a representative sample from 13 countries. Comput. Hum. Behav. 27, 585–589. doi: 10.1016/j.chb.2010.10.009

[ref2] Andreassen C. S. Torsheim T. Brunborg G. S. Pallesen S. (2012). Development of a Facebook addiction scale. Psychol. Rep. 110, 501–517. doi: 10.2466/2F02.09.18.PR0.110.2.501-517 22662404

[ref3] Barnum D. D. Snyder C. R. Rapoff M. A. Mani M. M. Thompson R. (1998). Hope and social support in psychological adjustment of children who have survived burn injuries and their matched controls. Child. Health Care 27, 15–30. doi: 10.1207/s15326888chc2701_2

[ref4] Belfort E. L. Miller L. (2018). Relationship between adolescent suicidality, self-injury, and media habits. Child Adolesc. Psych. Clin. 27, 159–169. doi: 10.1016/j.chc.2017.11.004, PMID: 29502743

[ref5] Brage D. Meredith W. M. Woodward J. (1993). Correlates of loneliness among Midwestern adolescents. Adolescence 28, 685–693. doi: 10.1016/0306-4603(93)90075-K, PMID: 8237553

[ref6] Brailovskaia J. Rohmann E. Bierhoff H.-W. Schillack H. Margraf J. (2019). The relationship between daily stress, social support and Facebook addiction disorder. Psychiatry Res. 276, 167–174. doi: 10.1016/j.psychres.2019.05.014, PMID: 31096147

[ref7] Bronfenbrenner U. (1979). The Ecology of Human Development. Cambridge: Harvard University Press.

[ref8] Caplan S. E. (2003). Preference for online social interaction: a theory of problematic internet use and psychosocial well-being. Commun. Res. 30, 625–648. doi: 10.1177/0093650203257842

[ref9] Carroll N. Conboy K. (2020). Normalising the new normal: changing tech-driven work practices under pandemic time pressure. Int. J. Inf. Manag. 55, 102–186. doi: 10.1016/j.ijinfomgt.2020.102186, PMID: 32836643 PMC7358767

[ref10] Casale S. Fioravanti G. (2011). Psychosocial correlates of internet use among Italian students. Int. J. Psychol. 46, 288–298. doi: 10.1080/00207594.2010.541256, PMID: 22044272

[ref11] Chen Y. (2019). How does communication anxiety influence well-being? Examining the mediating roles of preference for online social interaction (POSI) and loneliness. Int. J. Commun. 13:19.

[ref12] China Internet network information center (2007). The 20th China statistical report on the internet development. Available at: http://www.cnnic.net.cn/NMediaFile/2022/0830/MAIN1661849329404PZVE2C5OXY.pdf (Accessed December 10, 2022).

[ref13] China Internet network information center (2022). The 50th China statistical report on the internet development. Available at: http://www.cnnic.net.cn/hlwfzyj/hlwxzbg/hlwtjbg/202209/P020220916626882289134.pdf (Accessed December 10, 2022).

[ref14] Chopik W. J. (2016). The benefits of social technology use among older adults are mediated by reduced loneliness. Cyberpsychol. Behav. Soc. Netw. 19, 551–556. doi: 10.1089/cyber.2016.0151, PMID: 27541746 PMC5312603

[ref15] Cunningham K. B. Kroll T. Wells M. (2021). First steps in identifying and addressing loneliness in the context of COVID-19. Perspect. Public Health 141, 200–201. doi: 10.1177/1757913920975793, PMID: 33629619 PMC8295934

[ref16] Deng S. S. Wang X. Y. (2005). Impact of internet factors on college Students' depression and loneliness. J. Hangzhou Teach. Coll. 25, 426–428.

[ref17] Deutrom J. Katos V. Al-Mourad M. B. Ali R. (2022). The relationships between gender, life satisfaction, loneliness and problematic internet use during CoViD-19: does the lockdown matter? Int. J. Environ. Res. Public Health 19:1325. doi: 10.3390/ijerph19031325, PMID: 35162348 PMC8835331

[ref18] Ding D. Q. (2003). Interpersonal interaction in cyberspace: a theoretical and empirical study. Doctoral dissertation Nanjing Normal University.

[ref19] Dwivedi Y. K. Hughes D. L. Coombs C. Constantiou I. Upadhyay N. (2020). Impact of covid-19 pandemic on information management research and practice: transforming education, work and life. Int. J. Inf. Manag. 55:102211. doi: 10.1016/j.ijinfomgt.2020.102211

[ref20] Ellwardt L. Aartsen M. Deeg D. Steverink N. (2013). Does loneliness mediate the relation between social support and cognitive functioning in later life? Soc. Sci. Med. 98, 116–124. doi: 10.1016/j.socscimed.2013.09.002, PMID: 24331889

[ref21] Falgoust G. Winterlind E. Moon P. Parker A. Zinzow H. Madathil K. C. (2022). Applying the uses and gratifications theory to identify motivational factors behind young adult's participation in viral social media challenges on TikTok. Hum. Fact. Healthc. 2:100014. doi: 10.1016/j.hfh.2022.100014

[ref22] Fang J. Wang X. Wen Z. Zhou J. (2020). Fear of missing out and problematic social media use as mediators between emotional support from social media and phubbing behavior. Addict. Behav. 107:106430. doi: 10.1016/j.addbeh.2020.106430, PMID: 32289745

[ref23] Fernandes B. Uzun B. Aydin C. Tan-Mansukhani R. Vallejo A. Saldaña-Gutierrez A.. (2021). Internet use during COVID-19 lockdown among young people in low-and middle-income countries: role of psychological wellbeing. Addict. Behav. Rep. 14:100379. doi: 10.1016/j.abrep.2021.100379, PMID: 34608443 PMC8481788

[ref24] Gilmour J. Machin T. Brownlow C. Jeffries C. (2019). Facebook-based social support and health: a systematic review. Psychol. Pop. Media Cult. 9, 328–346. doi: 10.1037/ppm0000246

[ref25] Gökhan B. (2010). An investigation of the relationship between shyness and loneliness levels of elementary students in a Turkish sample. Int. Online J. Educ. Sci. 2, 419–440.

[ref26] Goncalves G. Oliveira T. Cruz-Jesus F. (2018). Understanding individual-level digital divide: evidence of an African country. Comput. Hum. Behav. 87, 276–291. doi: 10.1016/j.chb.2018.05.039

[ref27] Greenberg J. Solomon S. Pyszczynski T. Rosenblatt A. Burling J. Lyon D.. (1992). Why do people need self-esteem? Converging evidence that self-esteem serves an anxiety-buffering function. J. Pers. Soc. Psychol. 63, 913–922. doi: 10.1037/0022-3514.63.6.913, PMID: 1460559

[ref28] Greenstein S. (2020). The basic economics of internet infrastructure. J. Econ. Perspect. 34, 192–214. doi: 10.1257/jep.34.2.192

[ref29] Groarke J. M. Berry E. Graham-Wisener L. McKenna-Plumley P. E. McGlinchey E. Armour C. (2020). Loneliness in the UK during the COVID-19 pandemic: cross-sectional results from the COVID-19 psychological wellbeing study. PLoS One 15:e0239698. doi: 10.1371/journal.pone.0239698, PMID: 32970764 PMC7513993

[ref30] Guessoum S. B. Lachal J. Radjack R. Carretier E. Minassian S. Benoit L.. (2020). Adolescent psychiatric disorders during the COVID-19 pandemic and lockdown. Psychiatry Res. 291:113264. doi: 10.1016/j.psychres.2020.113264, PMID: 32622172 PMC7323662

[ref31] Haferkamp N. Krämer N. C. (2011). Social comparison 2.0: examining the effects of online profiles on social-networking sites. Cyberpsychol. Behav. Soc. Netw. 14, 309–314. doi: 10.1089/cyber.2010.0120, PMID: 21117976

[ref32] Harter S. (1993). “Causes and consequences of low self-esteem in children and adolescents,” in Self-esteem: The Puzzle of Low Self-regard. ed. Baumeister R. F. (New York: Plenum Press).

[ref33] Hayes A. F. (2017). Introduction to Mediation, Moderation, and Conditional Process Analysis: A Regression-based Approach. New York: Guilford publications.

[ref34] He H. L. (2018). A study on the questionnaire development of online social preference and its relationship with loneliness and two types of social support Master dissertation Guangzhou University

[ref35] Ho H. Ito K. (2019). Consumption-oriented engagement in social network sites: undesirable influence on personal well-being. Eur. J. Mark. 53, 1355–1377. doi: 10.1108/EJM-11-2017-0809

[ref36] Holmes E. A. O'Connor R. C. Perry V. H. Tracey I. Wessely S. Arseneault L.. (2020). Multidisciplinary research priorities for the COVID-19 pandemic: a call for action for mental health science. Lancet Psych. 7, 547–560. doi: 10.1016/S2215-0366(20)30168-1, PMID: 32304649 PMC7159850

[ref37] Huang Y. R. (2018). A study on the relationship between high school students' personality, cell phone use behavior preference and loneliness Master dissertation Qinghai Normal University.

[ref38] Jackson T. Soderlind A. Weiss K. E. (2000). Personality traits and quality of relationships as predictors of future loneliness among American college students. Soc. Behav. Personal. Int. J. 28, 463–470. doi: 10.2224/sbp.2000.28.5.463

[ref39] Jeste D. V. Lee E. E. Cacioppo S. (2020). Battling the modern behavioral epidemic of loneliness: suggestions for research and interventions. JAMA Psychiat. 77, 553–554. doi: 10.1001/jamapsychiatry.2020.0027, PMID: 32129811 PMC7483387

[ref40] Jia Z. Wang Y. Yang Y. Yang L. (2018). Chinese university students' loneliness and generalized pathological internet use: a longitudinal cross-lagged analysis. Soc. Behav. Personal. Int. J. 46, 861–870. doi: 10.2224/sbp.6807

[ref41] Katz E. Blumler J. G. (1974). The Uses of Mass Communications: Current Perspectives on Gratifications Research. Beverly Hills: Sage Publications.

[ref42] Kaynar O. Amichai-Hamburger Y. (2008). The effects of need for cognition on internet use revisited. Comput. Hum. Behav. 24, 361–371. doi: 10.1016/j.chb.2007.01.033

[ref43] King D. L. Delfabbro P. H. Billieux J. Potenza M. N. (2020). Problematic online gaming and the covid-19 pandemic. J. Behav. Addict. 9, 184–186. doi: 10.1556/2006.2020.00016, PMID: 32352927 PMC8939428

[ref44] Kola L. (2020). Global mental health and covid-19. Lancet Psychiatry 7, 655–657. doi: 10.1016/S2215-0366(20)30235-2, PMID: 32502468 PMC7266571

[ref45] Kong F. You X. (2013). Loneliness and self-esteem as mediators between social support and life satisfaction in late adolescence. Soc. Indic. Res. 110, 271–279. doi: 10.1007/s11205-011-9930-6

[ref46] Kraut R. Patterson M. Lundmark V. Kiesler S. Scherlis W. (1998). Internet paradox. A social technology that reduces social involvement and psychological well-being? Am. Psychol. 53, 1017–1031. doi: 10.1037//0003-066X.53.9.1017, PMID: 9841579

[ref47] Lakey B. Cohen S. (2000). “Social support theory and measurement,” in Social Support Measurement and Intervention: A Guide for Health and Social Scientists. eds. Cohen S. Underwood L. G. Gottlieb B. H. (Oxford: Oxford University Press).

[ref48] Leary M. R. Tambor E. S. Terdal S. K. Downs D. L. (1995). Self-esteem as an interpersonal monitor: the sociometer hypothesis. J. Pers. Soc. Psychol. 68, 518–530. doi: 10.1037/0022-3514.68.3.518

[ref49] Lee H. E. Cho J. (2019). Social media use and well-being in people with physical disabilities: influence of SNS and online community uses on social support, depression, and psychological disposition. Health Commun. 34, 1043–1052. doi: 10.1080/10410236.2018.1455138, PMID: 29652521

[ref50] Lei L. Liu M. X. (2005). The relationship of Adolescents' personality with their using social Service of Internet. Acta Psychol. Sin. 37, 797–802. doi: 10.3969/j.issn.1001-4918.2005.04.008

[ref51] Leung L. (2011). Loneliness, social support, and preference for online social interaction: the mediating effects of identity experimentation online among children and adolescents. Chin. J. Commun. 4, 381–399. doi: 10.1080/17544750.2011.616285

[ref52] Li G. Y. Zhou Z. K. Ping F. (2012). Relationship between network well-being and the preference of internet contents among college students. Stud. Psychol. Behav. 10, 395–400.

[ref53] Li J. Han X. Wang W. Sun G. Cheng Z. (2018). How social support influences university students' academic achievement and emotional exhaustion: the mediating role of self-esteem. Learn. Individ. Diff. 61, 120–126. doi: 10.1016/j.lindif.2017.11.016

[ref54] Liang X. Y. (2008). A study on the effect mechanism of online social support on adolescents’ mental health. Doctoral dissertation China Central Normal University.

[ref55] Lin S. Liu D. Niu G. Longobardi C. (2022). Active social network sites use and loneliness: the mediating role of social support and self-esteem. Curr. Psychol. 41, 1279–1286. doi: 10.1007/s12144-020-00658-8

[ref56] Liu G. Li S. Kong F. (2022). Association between social support, smartphone usage and loneliness among the migrant elderly following children in Jinan, China: a cross-sectional study. BMJ Open 12:e060510. doi: 10.1136/bmjopen-2021-060510, PMID: 35613788 PMC9174823

[ref57] Liu M. X. (2006). The relationship between adolescents' personality traits, social support, and internet use preferences Doctoral dissertation Capital Normal University.

[ref58] Marsden J. Darke S. Hall W. Hickman M. Holmes J. Humphreys K.. (2020). Mitigating and learning from the impact of COVID-19 infection on addictive disorders. Addiction 115, 1007–1010. doi: 10.1111/add.15080, PMID: 32250482 PMC9364227

[ref59] Mestre-Bach G. Blycker G. R. Potenza M. N. (2020). Pornography use in the setting of the COVID-19 pandemic. J. Behav. Addict. 9, 181–183. doi: 10.1556/2006.2020.00015, PMID: 32663384 PMC8939420

[ref60] Mickelson K. D. (1997). Seeking Social Support: Parents in Electronic Support Groups Culture of the Internet. Mahwah: Lawrence Erlbaum Associates.

[ref61] Mitchell W. S. (1999). Social and Psychological Factors Associated with Internet Use in the Home: A Uses and Gratifications Study. Bowling Green: Bowling Green State University.

[ref500] Morgan C. Cotten S. R. (2003). The relationship between internet activities and depressive symptoms in a sample of college freshmen. Cyberpsychol. Behav. 6, 133–142. doi: 10.1089/109493103321640329 12804025

[ref62] Ni X. L. Shao X. Y. (2019). Influence of Adolescents' use of online social media on their sense of happiness: serial mediating paths of self-esteem linking self-identity. J. Lanzhou Univ. 47, 122–133.

[ref63] Nie N. H. (2001). Sociability, interpersonal relations, and the internet: reconciling conflicting findings. Am. Behav. Sci. 45, 420–435. doi: 10.1177/00027640121957

[ref64] Orth U. Robins R. W. Meier L. L. Conger R. D. (2016). Refining the vulnerability model of low self-esteem and depression: disentangling the effects of genuine self-esteem and narcissism. J. Pers. Soc. Psychol. 110, 133–149. doi: 10.1037/PSPP0000038, PMID: 25915133

[ref65] Pai N. Vella S. L. (2021). COVID-19 and loneliness: a rapid systematic review. Aust. N. Z. J. Psych. 55, 1144–1156. doi: 10.1177/0004867421103148, PMID: 34256632

[ref66] Parker J. S. Benson M. J. (2004). Parent-adolescent relations and adolescent functioning: self-esteem, substance abuse, and delinquency. Adolescence 39, 519–530. 15673227

[ref67] Perlman D. Peplau L. A. (1981). Toward a social psychology of loneliness. Pers. Relat. 3, 31–56.

[ref68] Romer D. Bagdasarov Z. More E. (2013). Older versus newer media and the well-being of United States youth: results from a national longitudinal panel. J. Adolesc. Health 52, 613–619. doi: 10.1016/j.jadohealth.2012.11.012, PMID: 23375827

[ref69] Rosenberg M. (1965). Self-esteem and the adolescent. Economics and the social sciences: society and the adolescent self-image. N. Engl. Q. 67, 177–196. doi: 10.1353/tj.2015.0042

[ref70] Rosenberg M. (2015). Society and the adolescent self-image: Selection of the sample. Princeton University Press.

[ref71] Salemink K. Strijker D. Bosworth G. (2017). Rural development in the digital age: a systematic literature review on unequal ICT availability, adoption, and use in rural areas. J. Rural. Stud. 54, 360–371. doi: 10.1016/j.jrurstud.2015.09.001

[ref72] Selfhout M. H. W. Branje S. J. T. Delsing M. ter Bogt T. F. M. Meeus W. H. J. (2009). Different types of internet use, depression, and social anxiety: the role of perceived friendship quality. J. Adolesc. 32, 819–833. doi: 10.1016/j.adolescence.2008.10.011, PMID: 19027940

[ref73] Shaw L. H. Gant L. M. (2002). In defense of the internet: the relationship between internet communication and depression, loneliness, self-esteem, and perceived social support. Cyberpsychol. Behav. 5, 157–171. doi: 10.1089/109493102753770552, PMID: 12025883

[ref74] Shen G. (2013). A study on the relationship between SoLoMo interaction behavior and loneliness and happiness among college students Master dissertation Southwest University.

[ref75] Shen C. X. Liu R. D. Wang D. (2013). The relationship between internet use and Children's loneliness--a moderating effect of personality. J. Psychol. Sci. 5, 1140–1145.

[ref76] Shi Y. Luo Y. L. Liu Y. Yang Z. (2019). Affective experience on social networking sites predicts psychological well-being off-line. Psychol. Rep. 122, 1666–1677. doi: 10.1177/0033294118789039, PMID: 30080110

[ref77] Shovestul B. Han J. Germine L. Dodell-Feder D. (2020). Risk factors for loneliness: the high relative importance of age versus other factors. PLoS One 15:e0229087. doi: 10.1371/journal.pone.0229087, PMID: 32045467 PMC7012443

[ref78] Singh S. Pandeya N. M. Datta M. Batra S. (2021). Stress, internet use, substance use and coping among adolescents, young-adults and middle-age adults amid the 'new normal' pandemic era. Clin. Epidemiol. Glob. Health 12:100885. doi: 10.1016/j.cegh.2021.100885 PMC10250820 37309373

[ref79] Sipowicz K. Podlecka M. Mokros Ł. Pietras T. (2021). Lonely in the City–sociodemographic status and somatic morbidities as predictors of loneliness and depression among seniors–preliminary results. Int. J. Environ. Res. Public Health 18:7213. doi: 10.3390/ijerph18147213, PMID: 34299666 PMC8305915

[ref80] Sira N. Ballard S. M. (2009). An ecological approach to examining body satisfaction in Caucasian and African American female college students. Fam. Consum. Sci. Res. J. 38, 208–226. doi: 10.1111/j.1552-3934.2009.00021.x

[ref81] Skues J. L. Williams B. Wise L. (2012). The effects of personality traits, self-esteem, loneliness, and narcissism on Facebook use among university students. Comput. Hum. Behav. 28, 2414–2419. doi: 10.1016/j.chb.2012.07.012

[ref82] Smith B. J. Lim M. H. (2020). How the COVID-19 pandemic is focusing attention on loneliness and social isolation. Public Health Res. Pract. 30:3022008. doi: 10.17061/phrp3022008, PMID: 32601651

[ref83] Stockwell S. Stubbs B. Jackson S. E. Fisher A. Yang L. Smith L. (2021). Internet use, social isolation and loneliness in older adults. Age. Soc. 41, 2723–2746. doi: 10.1017/S0144686X20000550

[ref84] Sun Y. Li Y. Bao Y. Meng S. Sun Y. Schumann G.. (2020). Brief report: increased addictive internet and substance use behavior during the COVID-19 pandemic in China. Am. J. Addict. 29, 268–270. doi: 10.1111/ajad.13066, PMID: 32500608 PMC7300868

[ref85] Swickert R. J. Hittner J. B. Harris J. L. Herring J. A. (2002). Relationships among internet use, personality, and social support. Comput. Hum. Behav. 18, 437–451. doi: 10.1016/S0747-5632(01)00054-1

[ref86] Tajfel H. Turner J. C. (1986). “The social identity theory of intergroup behavior,” in Psychology of Intergroup Relations. eds. Worchel S. Austin W. (Chicago: Nelson Hall).

[ref87] Taquet M. Luciano S. Geddes J. R. Harrison P. J. (2021). Bidirectional associations between COVID-19 and psychiatric disorder: retrospective cohort studies of 62 354 COVID-19 cases in the USA. Lancet Psychiatry 8, 130–140. doi: 10.1016/S2215-0366(20)30462-4, PMID: 33181098 PMC7820108

[ref102] Tian Y. Qin N. Cao S. Gao F. (2021). Reciprocal associations between shyness, self-esteem, loneliness, depression and internet addiction in Chinese adolescents. Addict. Res. Theory 29, 98–110. doi: 10.1080/16066359.2020.1755657

[ref88] Torales J. M O’Higgins M. Castaldelli-Maia J. M. Ventriglio A. (2020). The outbreak of COVID-19 coronavirus and its impact on global mental health. Int. J. Soc. Psychiatry 66, 317–320. doi: 10.1177/0020764020915212 32233719

[ref89] Trepte S. Dienlin T. Reinecke L. (2015). Influence of social support received in online and offline contexts on satisfaction with social support and satisfaction with life: a longitudinal study. Media Psychol. 18, 74–105. doi: 10.1080/15213269.2013.838904

[ref90] Tsai C. Y. (2022). Social support, self-esteem, and levels of stress, depression, and anxiety during the COVID-19 pandemic. Scripps Senior Theses. Available at: https://scholarship.claremont.edu/scripps_theses/1789 (Accessed December 10, 2022).

[ref91] Uchino B. N. Cacioppo J. T. Kiecolt Glaser J. K. (1996). The relationship between social support and physiological processes: a review with emphasis on underlying mechanisms and implications for health. Psychol. Bull. 119, 488–531. doi: 10.1037/0033-2909.119.3.488, PMID: 8668748

[ref92] Valkenburg P. M. Peter J. Schouten A. P. (2006). Friend networking sites and their relationship to adolescents' well-being and social self-esteem. Cyberpsychol. Behav. 9, 584–590. doi: 10.1089/cpb.2006.9.584, PMID: 17034326

[ref93] Vanhalst J. Luyckx K. Scholte R. H. Engels R. C. Goossens L. (2013). Low self-esteem as a risk factor for loneliness in adolescence: perceived-but not actual-social acceptance as an underlying mechanism. J. Abnorm. Child Psychol. 41, 1067–1081. doi: 10.1007/s10802-013-9751-y 23661185

[ref94] Wallinheimo A. S. Evans S. L. (2022). Patterns of internet use, and associations with loneliness, amongst middle-aged and older adults during the COVID-19 pandemic. Healthcare 10:. doi:10.3390/healthcare10071179 35885706 PMC9324051

[ref95] Wang K. (2018). A meta-analysis of relationship between college students’ self-esteem and social support in China. Chin. J. Health Psychol. 26:5. doi: 10.13342/j.cnki.cjhp.2018.06.035

[ref96] Wang X. D. Wang X. L. Ma H. (1999). Mental health rating scale updated version. Beijing: China Journal of Mental Health, Inc.

[ref97] Weiser E. B. (2001). The functions of internet use and their social and psychological consequences. Cyberpsychol. Behav. 4, 723–743. doi: 10.1089/109493101753376678 11800180

[ref98] Weiss R. S. (1974). “The provisions of social relationships,” in Doing Unto Others. ed. Rubin Z. (Englewood Cliffs, NJ: Prentice-Hall).

[ref99] Yang Q. Ye B. J. (2014). The effect of gratitude on Adolescents' life satisfaction: the mediating role of perceived social support and the moderating role of stressful life events. J. Psychol. Sci. 37, 610–616.

[ref100] Ye T. (2019). The effect of social networking site usage intensity on loneliness: The mediating role of social comparison and self-esteem Master dissertation Shandong Normal University.

[ref101] Ye Y. Lin L. (2015). Examining relations between locus of control, loneliness, subjective well-being, and preference for online social interaction. Psychol. Rep. 116, 164–175. doi: 10.2466/07.09.PR0.116k14w3, PMID: 25621672

[ref103] Zandifar A. Badrfam R. (2020). Iranian mental health during the COVID-19 epidemic. Asian J. Psychiatr. 51:101990. doi: 10.1016/j.ajp.2020.101990, PMID: 32163908 PMC7128485

[ref104] Zhang H. Peng S. Li S. Li J. Yu Q. (2022). Online social support and depressive symptoms: mediating effect of self-esteem and gender differences. Int. J. Ment. Heal. Addict., 1–14. doi: 10.1007/s11469-022-00818-w

[ref105] Zhang X. K. Zhang L. Zhao Y. Q. (2004). Theory constructing about the self-esteem structural model. J. Psychol. Sci. 4, 791–795. doi: 10.3969/j.issn.1671-6981.2004.04.006

[ref106] Zhang Z. T. Wang J. Q. Liu F. (2012). Relationship between parenting styles, perceived social support, loneliness and subjective well-being of undergraduates. China. J. Health Psychol. 20, 1080-1081–1080-1083.

[ref107] Zhou Z. Cheng Q. (2022). Relationship between online social support and adolescents’ mental health: a systematic review and meta-analysis. J. Adolesc. 94, 281–292. doi: 10.1002/jad.12031, PMID: 35390193

[ref108] Zhou H. Long L. R. (2004). Statistical remedies for common method biases. Adv. Psychol. Sci. 12, 942–950. doi: 10.3969/j.issn.1671-3710.2004.06.018

[ref109] Zhou Z. K. Wang C. Q. (2015). Does cyber socializing increase loneliness. J. Suzhou Univ. 3, 81–91.

